# Gene expression profiling in blood from cerebral malaria patients and mild malaria patients living in Senegal

**DOI:** 10.1186/s12920-019-0599-z

**Published:** 2019-10-30

**Authors:** Alassane Thiam, Michel Sanka, Rokhaya Ndiaye Diallo, Magali Torres, Babacar Mbengue, Nicolas Fernandez Nunez, Fatou Thiam, Gora Diop, Geneviève Victorero, Catherine Nguyen, Alioune Dieye, Pascal Rihet

**Affiliations:** 1 Unité d’Immunogénétique, Institut Pasteur de Dakar, Dakar, Sénégal; 2 Aix Marseille Univ, INSERM, TAGC UMR U1090, 163 Av de Luminy, 13288 Marseille, cedex 9 France; 3 Service de Génétique Humaine, Faculté de Médecine, de Pharmacie et d’Odontostomatologie, UCAD, Dakar, Sénégal; 4 Service Immunologie, Faculte de Medecine, Université Cheikh Anta Diop de Dakar, Dakar, Sénégal; 5 Département de Génie chimique et biologie, Ecole Supérieure Polytechnique, Université Cheikh Anta Diop de Dakar, Dakar, Sénégal; 6 Département de Biologie animale, Faculté des Sciences et Techniques, Université Cheikh Anta Diop de Dakar, Dakar, Sénégal

**Keywords:** Transcriptome, Gene expression profiling, Cerebral malaria, Mild malaria

## Abstract

**Background:**

*Plasmodium falciparum* malaria remains a major health problem in Africa. The mechanisms of pathogenesis are not fully understood. Transcriptomic studies may provide new insights into molecular pathways involved in the severe form of the disease.

**Methods:**

Blood transcriptional levels were assessed in patients with cerebral malaria, non-cerebral malaria, or mild malaria by using microarray technology to look for gene expression profiles associated with clinical status. Multi-way ANOVA was used to extract differentially expressed genes. Network and pathways analyses were used to detect enrichment for biological pathways.

**Results:**

We identified a set of 443 genes that were differentially expressed in the three patient groups after applying a false discovery rate of 10%. Since the cerebral patients displayed a particular transcriptional pattern, we focused our analysis on the differences between cerebral malaria patients and mild malaria patients. We further found 842 differentially expressed genes after applying a false discovery rate of 10%. Unsupervised hierarchical clustering of cerebral malaria-informative genes led to clustering of the cerebral malaria patients. The support vector machine method allowed us to correctly classify five out of six cerebral malaria patients and six of six mild malaria patients. Furthermore, the products of the differentially expressed genes were mapped onto a human protein-protein network. This led to the identification of the proteins with the highest number of interactions, including GSK3B, RELA, and APP. The enrichment analysis of the gene functional annotation indicates that genes involved in immune signalling pathways play a role in the occurrence of cerebral malaria. These include BCR-, TCR-, TLR-, cytokine-, FcεRI-, and FCGR- signalling pathways and natural killer cell cytotoxicity pathways, which are involved in the activation of immune cells. In addition, our results revealed an enrichment of genes involved in Alzheimer’s disease.

**Conclusions:**

In the present study, we examine a set of genes whose expression differed in cerebral malaria patients and mild malaria patients. Moreover, our results provide new insights into the potential effect of the dysregulation of gene expression in immune pathways. Host genetic variation may partly explain such alteration of gene expression. Further studies are required to investigate this in African populations.

## Background

*Plasmodium falciparum* malaria remains a leading cause of mortality and morbidity in tropical countries. It encompasses a broad range of clinical phenotypes, including mild and severe forms of the disease. Cerebral malaria (CM), severe anaemia, and respiratory distress are the main syndromes of severe malaria (SM)*.* According to a World Health Organization (WHO) report, major complications account for 429,000 deaths annually [1]. In particular, CM is defined as a diffuse encephalopathy with seizures and impaired consciousness, and its case fatality rate has not changed over decades [2]. Patients are treated with antimalarial drugs, and it is thought that adjunctive therapies, such as anti-inflammatory, vasculo- and neuro-protective therapies, and new biomarkers for early diagnosis are needed. The outcome of infection depends on numerous factors, such as parasite virulence, host age, host immune status, and host and parasite genetics. Genome-wide association studies have been conducted in African populations to identify biomarkers associated with SM and to decipher the molecular basis of the pathogenesis. They have yielded, however, very few significant association results [3–5], likely due to a poor knowledge of the linkage disequilibrium patterns in African populations [6], and thus, to a lack of relevant tagSNPs for GWAS in Africa. Whole genome sequencing of individuals living in different African areas should overcome this limitation in the future. The assessment of genome-wide levels of expression is an alternative approach to identify relevant candidate genes and molecular pathways involved in pathogenesis.

In this way, transcriptional studies have been carried out in a CM mouse model, and have led to the discovery of genes, whose up- or down-regulation in several tissues was associated with CM, and which were involved in specific pathways, such as metabolic energy pathways, inflammatory response, antigen presentation, and T cell receptor signalling and pathways related to neurogenesis and neurodegenerative disorders [7, 8]. In humans, transcriptional profiling has been used very recently to search for molecular markers associated with malaria. Transcriptional studies have been carried out either in an in vitro model of CM or on blood cells taken from patients with either SM or mild malaria (MM). The in vitro model study has shown that TNF, platelets, and infected red blood cells significantly influence the expression of genes in endothelial cells [9]. Ex vivo studies that compared gene expression levels in SM patients and those in MM patients identified several host genes whose expression levels were associated with SM in Mali and Malawi [10–12], pointed out the involvement of immune pathways, such as interferon pathways and Toll-like receptor signalling [11, 12], and revealed genes involved in neurodegenerative disorders [13]. Moreover, a transcriptional study that investigated gene expression levels in blood taken from CM patients with and without malaria retinopathy revealed that cell adhesion and cytokine pathways were positively associated with malaria retinopathy [10]. In addition, ex vivo parasite transcriptional studies led to the identification of up- or down-regulated parasite genes associated with high parasitaemia or malaria retinopathy, and involved in cell adhesion, glycolysis or DNA replication [14, 15].

For the present study, we conducted a blood transcriptional analysis in Senegalese patients with CM, severe non-cerebral malaria (NCM), or MM in order to identify human genes whose expression levels are associated with the severity of the disease. We identified gene clusters that discriminated between CM and MM patients, and provided evidence of pathways associated with CM.

## Methods

### Patients and samples

Senegalese patients with clinical malaria were enrolled at the Principal Hospital of Dakar, the National Hospital Centre of Pikine, and the Regional Hospital of Tambacounda. Prior to enrolment, written or verbal information on the study was given in their native language, and informed consent was obtained from the participants or their relatives. At the day of admission, biological data including *P. falciparum* parasitaemia, haematological and biochemical characteristics were determined by hospitals’ medical laboratories and recorded, as previously described [16]. For all patients, the presence of *P. falciparum* infection was determined by an immunoassay detecting PfHRP2 (Standard diagnostics-Abott-Inc, Chicago, Illinois, USA). For 12 out of 16 patients *P. falciparum* parasitaemia was determined by microscopic examination of thin and thick blood smears, prior to anti-malarial treatment. The protocols were approved by the investigator’s institutions, the National Ethical Committee and the Ministry of Health of Senegal. The clinical cases were defined according to the WHO criteria [17]. MM cases were defined on the basis of fever with *P. falciparum* parasitaemia of < 25,000 parasites/μl of blood, with no evidence of impaired consciousness, convulsions, severe anaemia, hypoglycaemia, respiratory distress, or hypoxia. The presence of a deep coma, lack of purposeful response, lack of response to a painful stimulus by Glasgow score < 9, a diagnosed *P. falciparum* infection, without other clinically cause of impaired consciousness, such as hypoglycemia, meningitis, and encephalitis according to WHO criteria qualified the patients as having CM. NCM cases were defined on the basis of isolated symptoms of SM such as severe anaemia, hypoglycaemia, respiratory distress, or hypoxia, and the absence of neurological symptoms such as impaired consciousness, convulsions and long-term neurological deficits. Peripheral blood was taken by venipuncture on hospital admission. Blood samples (approximately 5 ml) were diluted and peripheral blood mononuclear cells (PBMC) were collected by Ficoll-Histopaque-1077 (Sigma-Aldrich, Saint-Quentin Fallavier) density-gradient centrifugation for RNA. After washing twice with PBS, PBMCs were stored at − 80 °C.

### Microarray processing (RNA extraction, amplification, and hybridization)

RiboNucleic Acid (RNA) extraction was carried out using TRIzol (Gibco, Invitrogen Corporation), according to the manufacturer’s instructions. The quantification of RNA was performed using the NanoDrop ND-1000 UV-Vis spectrophotometer (Thermo Fisher Scientific, Illkirch, France), and the quality of the RNA samples was analysed with the Bioanalyzer 2100 (Agilent Technologies®, Massy, France) and Agilent chips (RNA Nano Chip®). Samples with an RNA Integrity Number (RIN) > 8 were retained. Samples and microarrays were next processed according to Agilent’s recommendations. In brief, total RNA was reverse-transcribed with the AffinityScript RT enzyme and cDNA was transcribed in vitro. cRNA was further obtained using T7 RNA polymerase and labelled using Cy3-CTP, before fragmentation and hybridization (600 ng per sample for 17 h at 65 °C) to a SurePrint G3 Human GE 8x60k microarray. After washing, the microarrays were scanned with the SureScan Agilent Microarray Scanner.

### Microarray analysis

**Fig. 1:**
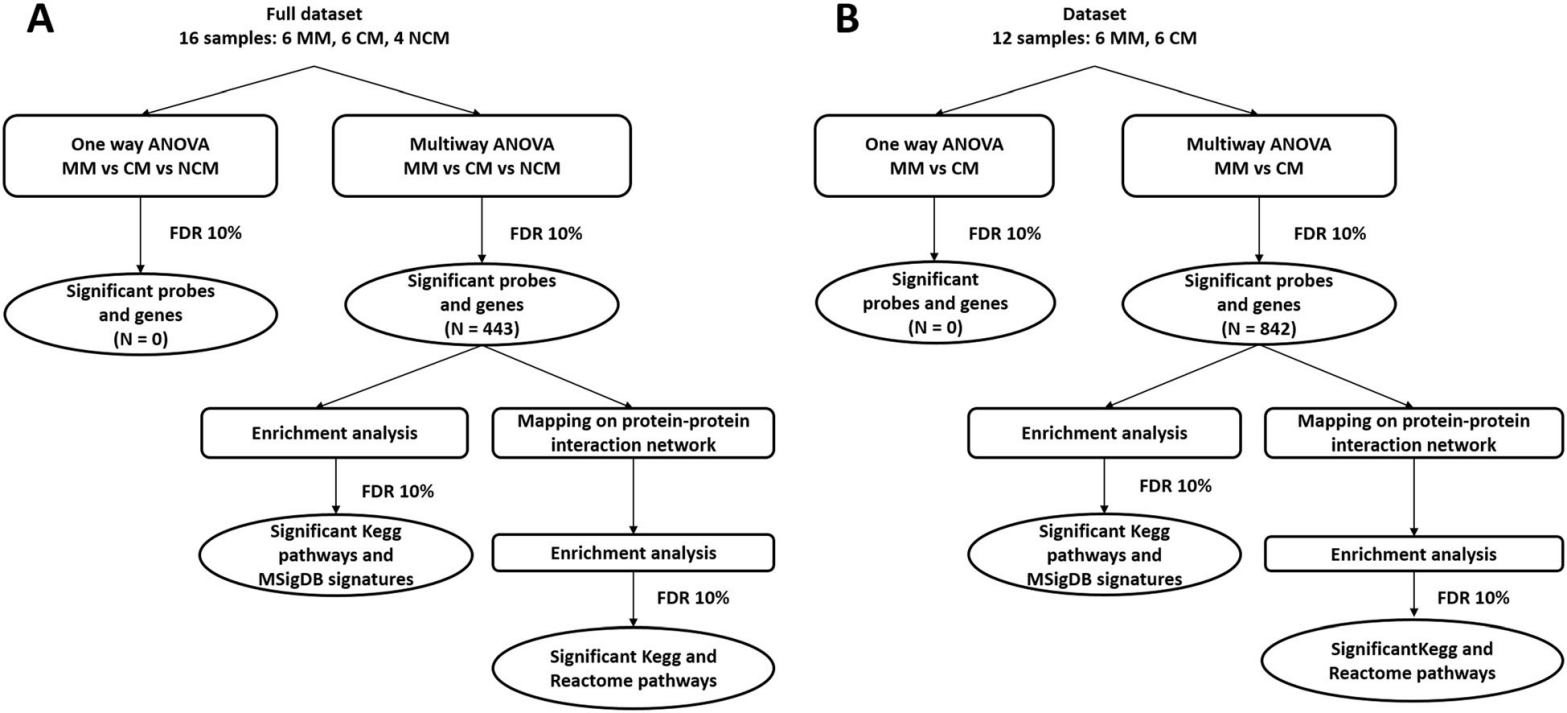
Schematic outline of statistical analyses. Blood gene expression levels in MM, CM, and NCM patients were compared (**a**), and blood gene expression levels in MM patients were compared to that in CM patients (**b**). Analyses of variance were carried out to identify significant probes and genes. Enrichment analyses were performed to determine molecular pathways potentially involved in malaria pathogenesis. An FDR of 10% was applied to correct for multiple tests

The quantification of signals was performed with Agilent’s Feature Extraction Software. From the raw data, data filtering, normalization and data backup were performed using the R statistical software package. The quantile method was used to normalize the data. A filter was applied on the raw data to delete controls, and then a second filter was applied to delete probes that showed a signal under the background; finally, 26,372 probes were further analyzed. Data were deposited in the GEO database under GSE116306. Statistical analysis was performed using the TIGR MeV (MultiExperiment Viewer) v4.1 software (http://mev.tm4.org/#/welcome) and the GeneANOVA program [18, 19]. Figure 1 shows the schematic outline of statistical analyses. Welch t-test was used to compare CM patient gene expression levels with those of MM patients. A multi-way ANOVA was used to take into account the influence of covariates, such as age, gender, and leukocyte counts. The ANOVA model gives an estimation of the contribution of each factor in the total variation of the whole data set. Furthermore, a local ANOVA allows the determination of each contribution for each gene; it gives an estimation of the variation due to the factor studied, and the significance of the estimate. An FDR of 10% was applied to correct for multiple tests. An unsupervised hierarchical clustering of the samples was carried out on the basis of the expression of the selected genes. A ‘1-out-iterative cross-validation’ testing procedure was used to ascertain the accuracy of the classification.

### Functional enrichment analysis

The gene annotation of the selected genes was performed using Genomic Regions Enrichment of Annotation Tool (GREAT), Enrichr, and NetworkAnalyst [20–22]. Those programs, which allow a biological interpretation of gene clusters on the basis of Molecular Signatures Database, Kyoto Encyclopaedia of Genes and Genomes (KEGG) pathways, Reactome pathways, and protein-protein interactions, were used to assess the functional enrichment for differentially expressed genes. Fisher exact and hypergeometric tests were used, whereas an FDR of 10% was applied to correct for multiple tests.

The selected genes were used to construct and visualize the network using NetworkAnalyst [21]. The underlying protein-protein interactions were obtained from InnateDB [23]. The minimum interaction network option was used, and a first-order interaction network that was composed only of the seed nodes and their direct interactors was created; it was further trimmed to keep only the nodes that connected the seed nodes. The degree centrality and the betweenness centrality were also calculated.

### Quantitative real time PCR

The RT-QPCR was performed on all samples from CM and MM patients. Eight genes (*ZBTB43, TICAM1, PI4K2A, HIC2, ZNHIT3, NFE2, NCR3* and *IL18R1*) were selected for validation experiments. RT-PCR was performed for each sample from the total RNA with the VILO SuperScript kit (Invitrogen, Carlsbad, California) based on the protocol provided. In summary, 500 ng of total RNA was mixed with 4 μl of the SuperScrip VILO MasterMix supplied in the kit and 5 pmol of primers in a total volume of 20 μl DEPC-treated water. The samples were vortexed and incubated, respectively, at 25 °C, 10 min and 42 °C, 1 h before the reaction was complete at 85 °C, 5 min. The qPCR testing was performed in 96-well plates with the product of the RT-PCR. Briefly, 2 μl of each sample, diluted to 1/10, was added to a solution containing 5 pmol / μl of each foward and reverse primer (Eurofins Genomics, Ebersberg), 12.5 μL of SYBR Green Master Mix (Thermo Fisher Scientific) and 8.5 μL of RNase / DNase free water. The experiment was carried out with the QuantStudio 6 Flex Real-Time PCR System apparatus using the following steps: pre-incubation cycle at 95 °C during 10 min; 40 amplification cycles at 95 °C during 15 s, and 60 °C during 60 s; 1 melting cycle at 95 °C during 15 s, 60 °C during 1 min, and 95 °C during 15 s. Gene expression levels were then calculated after normalizing the measurements on the GAPDH level and a reference sample.

## Results

### Patients

Blood samples taken from 16 patients were investigated: 6 from CM patients, 4 from severe NCM patients, and 6 from MM patients. Table 1 shows the patient characteristics in the patient groups. There was no significant difference between the groups for age (*P* = 0.15), haemoglobin concentration (*P* = 0.55), red blood cell count (*P* = 0.87), and leucocyte count (*P* = 0.81) on the basis of a Kruskal-Wallis test. In contrast, there was a difference for platelet count (*P* = 0.018).

**Table 1:** Characteristics of patients

	Cerebral malaria (*N* = 6)	Non cerebral malaria (*N* = 4)	Mild malaria (*N* = 6)
Gender			
Female	3	1	3
Male	3	3	3
Survival			
Dead	2	0	0
Alive	4	4	4
Age (years)			
Median (minimum-maximum)	21 (1–72)	3 (2–12)	3.5 (1–40)
Haemoglobin (g/dL)			
Median (minimum-maximum)	9.2 (7.9–15.1)	8.1 (3.2–14.1)	8.1 (3.7–12.6)
Red blood cells (×10^6^/μL)			
Median (minimum-maximum)	3.7 (2.8–8.4)	2.76 (1.36–3.9)	4.0 (1.6–4.7)
Leucocytes (× 10^3^/μL)			
Median (minimum-maximum)	13.5 (6.7–19.4)	10.1 (3.9–45.0)	15.0 (5.8–38.8)
Platelets (×10^3^/μL)			
Median (minimum-maximum)	53.5 (25.0–155.0)	287.0 (40.3–456.0)	309.0 (92.0–474.0)

### Gene expression analysis

**Fig. 2:**
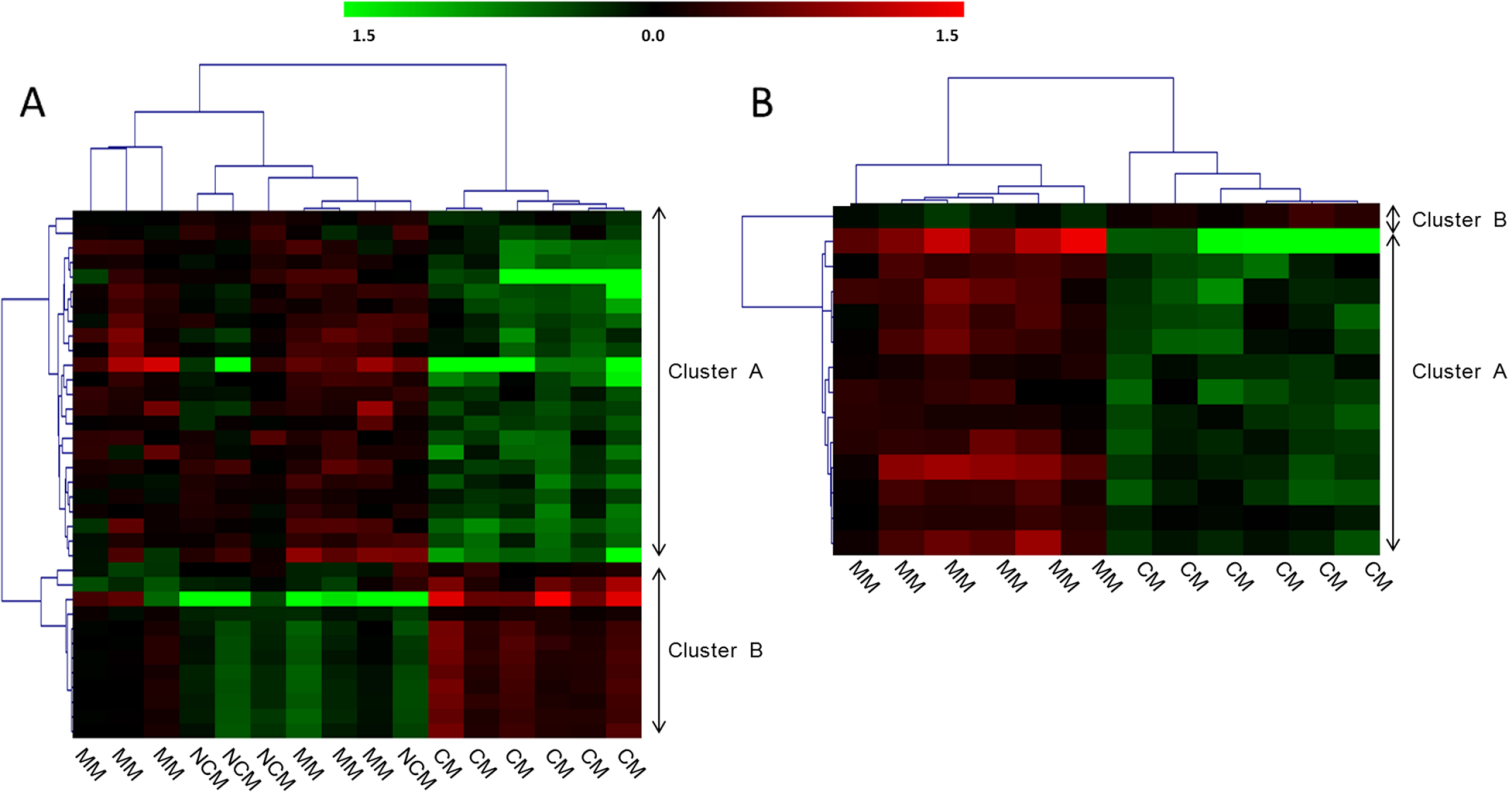
Gene expression profiles in blood samples from cerebral malaria (CM), severe non-cerebral malaria (NCM), and mild malaria (MM) patients. Red and green indicate expression levels above and below the median, respectively. A one-way ANOVA led to the selection of differentially expressed genes in the three patient groups, and those genes were used to carry out a hierarchical clustering (**a**). A Welch t-test led to the selection of 14 genes, and a hierarchical clustering was performed on this basis (**b**). Each column represents a patient, whereas each line represents a probe. Selected probes and genes had a nominal *P* value lower than 0.001. Genes that were down- and up-regulated in CM patients are shown in cluster A and cluster B, respectively. The list of those genes is shown in Additional file 1: Table S1

We performed a one-way ANOVA to identify genes whose expression may differ in MM, NCM, and CM patients. Thirty-six probes corresponding to 28 genes showed a difference with a nominal *P* value threshold of 0.001 (Additional file 1: Table S1), whereas there was no significant difference after applying a false discovery rate (FDR) of 10%. Since the CM patients appeared to show a particular pattern, we carried out a Welch t-test to compare the level of gene expression in CM patients with the level in MM patients. We identified 14 differentially expressed genes on the basis of nominal *P* values lower than 0.001 (Additional file 1: Table S1). However, after correcting for multiple tests, no significantly expressed genes remained. Figure 2 shows the unsupervised hierarchical clustering based on the 14 genes.

**Fig. 3:**
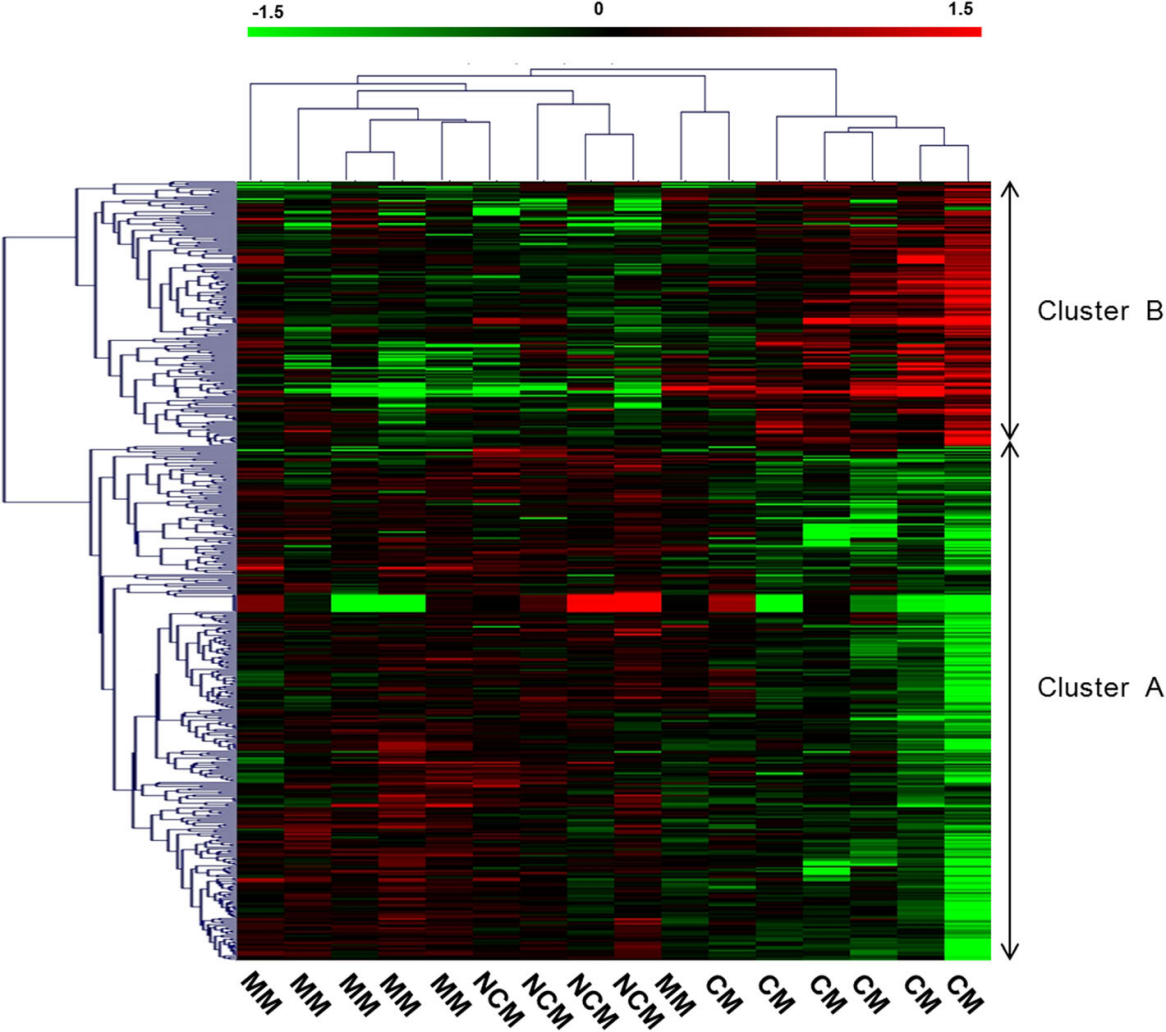
Hierarchical clustering of cerebral malaria (CM), severe non-cerebral (NCM) and mild malaria (MM) patients on the basis of differentially expressed genes. A multi-way ANOVA, which took into account confounding factors, led to the identification of genes whose expressions differed in CM, NCM, and MM patients after applying an FDR of 10%. Each column represents a patient. Genes that were down- and up-regulated in CM patients are shown in cluster A and cluster B, respectively. The list of those genes is shown in Additional file 1: Table S1

**Fig. 4:**
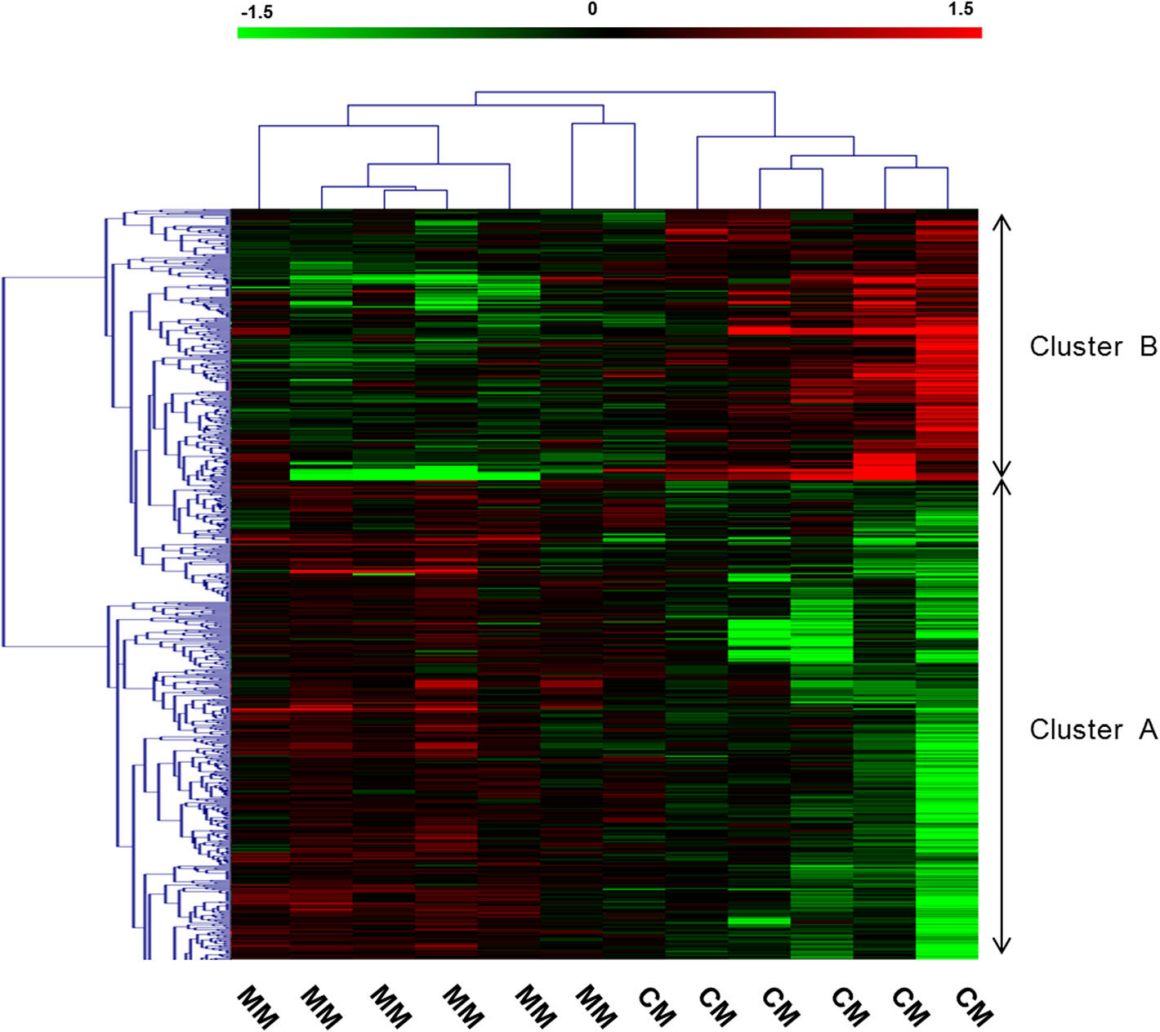
Hierarchical clustering of cerebral malaria (CM) and mild malaria (MM) patients on the basis of differentially expressed genes. A multi-way ANOVA was performed to selectively identify differentially expressed genes in CM and MM patients after an FDR of 10%. Each column represents a patient. Genes that were down- and up-regulated in CM patients are shown in cluster A and cluster B, respectively. The list of those genes is shown in Additional file 1: Table S1

We further performed a multi-way ANOVA to take into account the effect of potential confounding factors. First, we included MM, NCM, and CM patients, and then we considered only MM and CM patients. Tables 2 and 3 show the result of the global ANOVA; gene factor, gender, age, leukocyte count, and clinical status were sources of variation. Furthermore, we performed an ANOVA for each gene, taking into account the effect of confounding factors. The analysis of the three patient groups identified 503 probes corresponding to 443 genes whose expression levels differed significantly, with an FDR of 10% (Additional file 1: Table S1). For those genes, the median of the variance explained by clinical status was 19.6% (minimum = 4.7% and maximum = 36.2%). On the basis of the expression of those genes, the CM patients differed in their expression compared to MM and NCM patients (Fig. 3). We further investigated the pattern of gene expression in MM and CM patients. After applying an FDR of 10%, we identified 1060 probes corresponding to 842 genes, the expression of which differed in MM and CM (Additional file 1: Table S1). For those genes, the median of the variance explained by CM factor was 40.3% (minimum = 8.7% and maximum = 72.4%). The unsupervised hierarchical clustering of CM-informative genes is presented in Fig. 4. On this basis, the CM patients were clustered together, suggesting that gene expression profiles were well discriminated between cerebral and mild malaria patients. This was further supported by another analysis based on the SVM method. We used a ‘1-out-iterative cross-validation’ testing procedure. Iteratively, 1 out of the 12 samples was removed from the group and was classified based on the correlation between its expression profile and the median profile of samples from CM and MM patients. Accordingly, we correctly classified five out of six cerebral malaria patients and six of six mild malaria patients (*P* = 0.015).

**Table 2:** Multi-way ANOVA of gene expression levels in blood samples from MM, NCM, and CM patients

Factor	Sum of square	DF^a^	Variance	F	*P*-value
Gene	1,273,829,72	26,371	48,3	130,03	< 0,00001
Age	284,86	1	284,86	766,82	< 0,00001
Clinical status	1302,22	2	651,11	1752,7	< 0,00001
Gender	33,19	1	33,19	89,34	< 0,00001
Leucocyte	318,45	1	318,45	857,23	< 0,00001
Residual^b^	146,951,38	395,574	0,37	–	–
Total	1,422,740,94	421,951	3,37	–	–

^a^Degree of freedom

^b^The residual corresponds to the variance that was not explained by the model

**Table 3:** Multi-way ANOVA of gene expression levels in blood samples from MM and CM patients

Factor	Sum of square	DF^a^	Variance	F	*P*-value
Gene	1,149,572,54	24,575	46,78	135,88	< 0,00001
Age	330,03	1	330,03	958,71	< 0,00001
Cerebral malaria	1169,2	1	1169,2	3396,38	< 0,00001
Gender	152,75	1	152,75	443,71	< 0,00001
Leucocyte	147,91	1	147,91	429,65	< 0,00001
Residual^b^	126,902,45	368,636	0,34	–	–
Total	1,278,274,87	393,215	3,25	–	–

^a^Degree of freedom

^b^The residual corresponds to the variance that was not explained by the model

We further analysed the expression of eight selected genes by RT-qPCR to confirm their gene expression patterns. The level of gene expression measured with the microarray method was correlated with that measured with the qPCR method for the studied genes (Additional file 2: Figure S1). The correlation coefficient ranged from 0.69 to 0.94 (*P* < 0.02). Also, the qPCR results confirmed the microarray results.

### Functional enrichment

When analysing the genes showing a differential expressions in CM, NCM, and MM patients, there was no significant functional enrichment on the basis of the Kyoto Encyclopaedia of Genes and Genomes (KEGG) pathways. Nevertheless, the analysis of molecular immune signatures from the Molecular Signatures Database (MSigDB) showed an over-representation of genes regulated in activated CD4 lymphocytes, CD8 lymphocytes, B lymphocytes, NK lymphocytes, macrophages, monocytes, and dendritic cells (Table 4 and Additional file 3: Table S2). Using the differentially expressed genes in the CM and MM groups, we found very similar results (Table 5 and Additional file 3: Table S2).

**Table 4:** MsigDB molecular signatures enrichment analysis for genes differentially expressed between MM, CM and NCM patients

ID^a^	Description	*P* value	FDR
GSE29617	Genes up-regulated in comparison of peripheral blood mononuclear cells (PBMC) from TIV influenza vaccine pre-vaccination versus those at day 3 post-vaccination.	5,16E-07	9,85E-04
GSE17721	Genes up-regulated in comparison of dendritic cells (DC) stimulated with poly(I:C) (TLR3 agonist) at 1 h versus DC cells stimulated with Pam3Csk4 (TLR1/2 agonist) at 1 h.	9,56E-06	4,56E-03
GSE3337	Genes down-regulated in comparison of untreated CD8+ dendritic cells (DC) at 16 h versus those treated with IFNG [GeneID = 31,658] at 16 h.	2,75E-05	8,76E-03
GSE17721	Genes up-regulated in comparison of control dendritic cells (DC) at 4 h versus those stimulated with CpG DNA (TLR9 agonist) at 4 h.	5,22E-05	1,11E-02
GSE17721	Genes up-regulated in comparison of control dendritic cells (DC) at 2 h versus those stimulated with LPS (TLR4 agonist) at 2 h.	8,88E-05	1,54E-02
GSE19825	Genes down-regulated in comparison of naive CD8 T cells versus effector CD8 T cells.	1,34E-04	1,82E-02
GSE14308	Genes up-regulated in comparison of Th2 cells versus naive CD4 [GeneID = 620] T cells.	1,32E-04	1,94E-02
GSE17721	Genes up-regulated in comparison of dendritic cells (DC) stimulated with Gardiquimod (TLR7 agonist) at 0.5 h versus those stimulated with Gardiquimod (TLR7 agonist) at 8 h.	1,62E-04	2,07E-02
GSE9988	Genes down-regulated in comparison of monocytes treated with 5000 ng/ml LPS (TLR4 agonist) versus monocytes treated with control IgG.	2,38E-04	2,39E-02
GSE29617	Genes up-regulated in comparison of peripheral blood mononuclear cells (PBMC) from TIV influenza vaccinee before vaccination versus that after the vaccination.	2,70E-04	2,57E-02
GSE36476	Genes down-regulated in comparison of memory CD4 [GeneID = 920] T cells from young donors treated with TSST at 40 h versus those from old donors treated with TSST at 40 h.	4,19E-04	3,48E-02
GSE17721	Genes up-regulated in comparison of control dendritic cells (DC) at 24 h versus those stimulated with Pam3Csk4 (TLR1/2 agonist) at 24 h.	5,45E-04	4,00E-02
GSE9988	Genes down-regulated in comparison of monocytes treated with anti-TREM1 [GeneID = 54,210] and 5000 ng/ml LPS (TLR4 agonist) versus monocytes treated with control IgG.	5,41E-04	4,14E-02
GSE17721	Genes up-regulated in comparison of control dendritic cells (DC) at 12 h versus those stimulated with LPS (TLR4 agonist) at 12 h.	6,79E-04	4,32E-02
GSE25087	Genes down-regulated in comparison of fetal regulatory T cell (Treg) versus fetal conventional T cells.	2,69E-03	8,85E-02
GSE24634	Genes up-regulated in comparison of naive T cells at day 0 versus CD25+ regulatory T cell (Treg) treated with IL4 [GeneID = 3565] at day 7.	3,16E-03	9,73E-02

^a^MsigDB molecular signatures have been selected among those that were significant

**Table 5:** MsigDB molecular signatures enrichment analysis for genes differentially expressed between MM and CM patients

ID^a^	Description	*P* value	FDR
GSE14308	Genes up-regulated in comparison of Th2 cells versus naive CD4 [GeneID = 620] T cells.	2,98E-07	1,42E-04
GSE17721	Genes up-regulated in comparison of control dendritic cells (DC) at 12 h versus those stimulated with LPS (TLR4 agonist) at 12 h.	7,81E-06	1,07E-03
GSE14769	Genes up-regulated in comparison of unstimulated macrophage cells versus macrophage cells stimulated with LPS (TLR4 agonist) for 120 min.	7,52E-06	1,10E-03
GSE14000	Genes up-regulated in comparison of dendritic cells (DC) before and 4 h after LPS (TLR4 agonist) stimulation.	1,65E-05	1,75E-03
GSE11864	Genes up-regulated in comparison of untreated macrophages versus those cultured with M-CSF [GeneID = 1435] and IFNG [GeneID = 3458].	1,93E-05	1,76E-03
GSE18791	Genes up-regulated in comparison of control conventional dendritic cells (cDC) at 10 h versus cDCs infected with Newcastle disease virus (NDV) at 10 h.	1,04E-04	5,50E-03
GSE17974	Genes up-regulated in comparison of CD4 [GeneID = 920] T cells treated with IL4 [GeneID = 3565] and anti-IL12 at 0.5 h versus those at 72 h.	4,14E-04	1,47E-02
GSE15930	Genes down-regulated in comparison of CD8 T cells at 0 h versus those at 24 h after stimulation with IL12 .	5,62E-04	1,79E-02
GSE22886	Genes up-regulated in comparison of naive CD4 [GeneID = 920] T cells versus stimulated CD4 [GeneID = 920] Th2 cells at 12 h.	7,93E-04	2,13E-02
GSE25087	Genes down-regulated in comparison of fetal regulatory T cell (Treg) versus fetal conventional T cells.	1,45E-03	2,75E-02
GSE24634	Genes up-regulated in comparison of CD25+ T cells treated with IL4 [GeneID = 3565] versus CD25- T cells treated with IL4 [GeneID = 3565] at day 5.	1,69E-03	3,11E-02
GSE9988	Genes down-regulated in comparison of monocytes treated with anti-TREM1 [GeneID = 54,210] and 5000 ng/ml LPS (TLR4 agonist) versus monocytes treated with control IgG.	2,55E-03	3,77E-02
GSE14308	Genes down-regulated in comparison of Th1 cells versus induced regulatory T cell (Treg).	3,90E-03	4,81E-02
GSE13229	Genes down-regulated in comparison of mature NK cells versus intermediate mature NK cells.	4,43E-03	5,26E-02
GSE13306	Genes up-regulated in comparison of CD4 [GeneID = 920] T cells activated with lamina propria dendritic cells versus regulatory T cell (Treg).	7,03E-03	7,10E-02
GSE26495	Genes up-regulated in comparison of naive CD8 T cells versus PD-1 low CD8 T cells.	8,22E-03	7,74E-02
GSE17580	Genes down-regulated in comparison of regulatory T cell (Treg) from uninfected mice versus regulatory T cell (Treg) from mice infected with *S. mansoni*.	8,92E-03	7,85E-02
GSE14308	Genes up-regulated in comparison of Th2 cells versus natural regulatory T cell (Treg).	8,77E-03	7,86E-02
GSE22886	Genes up-regulated in comparison of naive CD4 [GeneID = 920] T cells versus stimulated CD4 [GeneID = 920] Th1 cells at 12 h.	1,04E-02	8,67E-02
GSE9650	Genes up-regulated in comparison of effector CD8 T cells versus exhausted CD8 T cells.	1,64E-05	1,84E-03
GSE25087	Genes down-regulated in comparison of fetal regulatory T cell (Treg) versus fetal conventional T cells.	1,45E-03	2,75E-02

^a^MsigDB molecular signatures have been selected among those that were significant

**Fig. 5:**
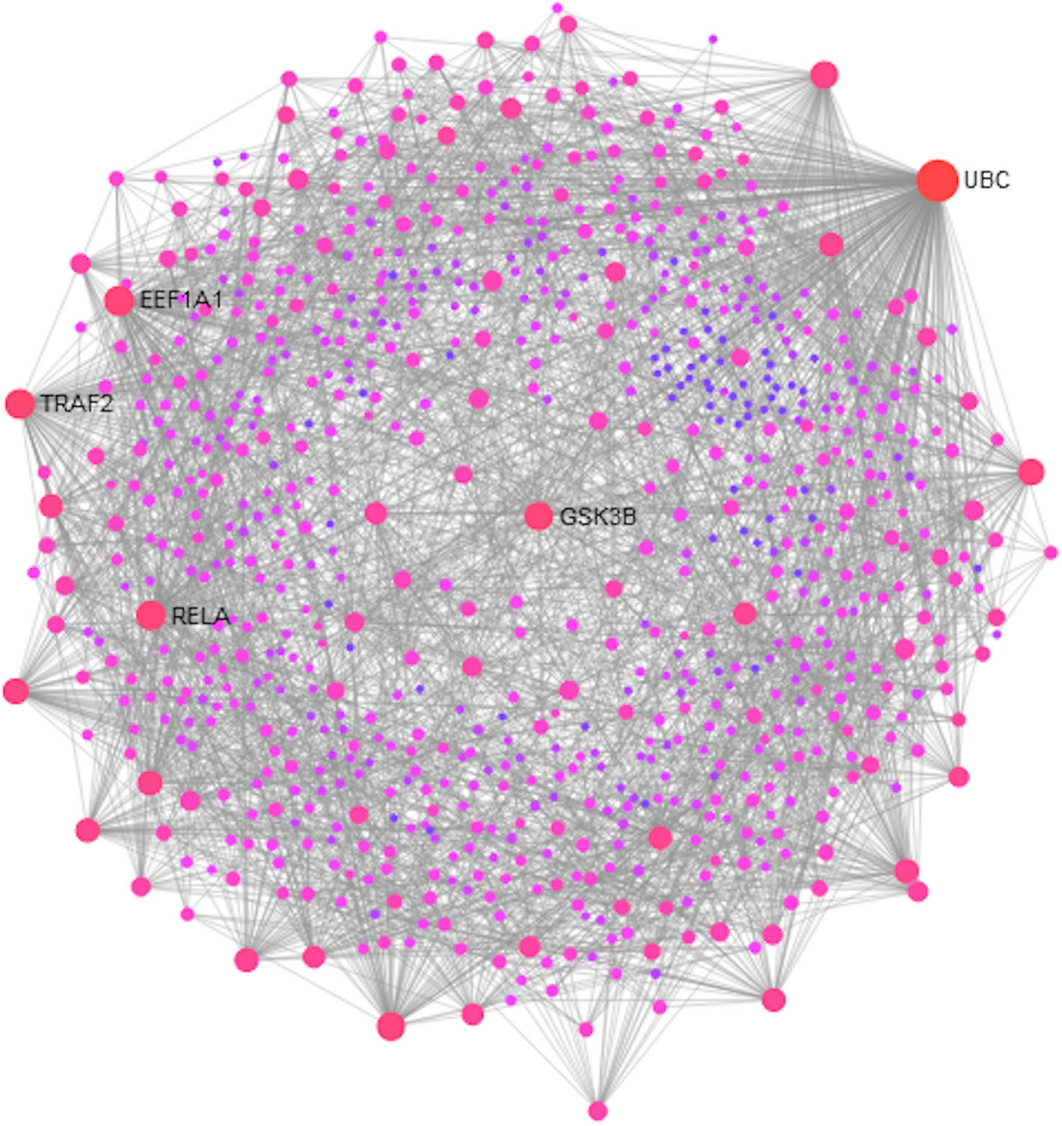
Protein-protein interaction (PPI) network of the differentially expressed genes in MM, CM, and CM patients. The size of the nodes is based on their degree centrality, whereas the intensity of their colour reflects their betweenness centrality

**Fig. 6:**
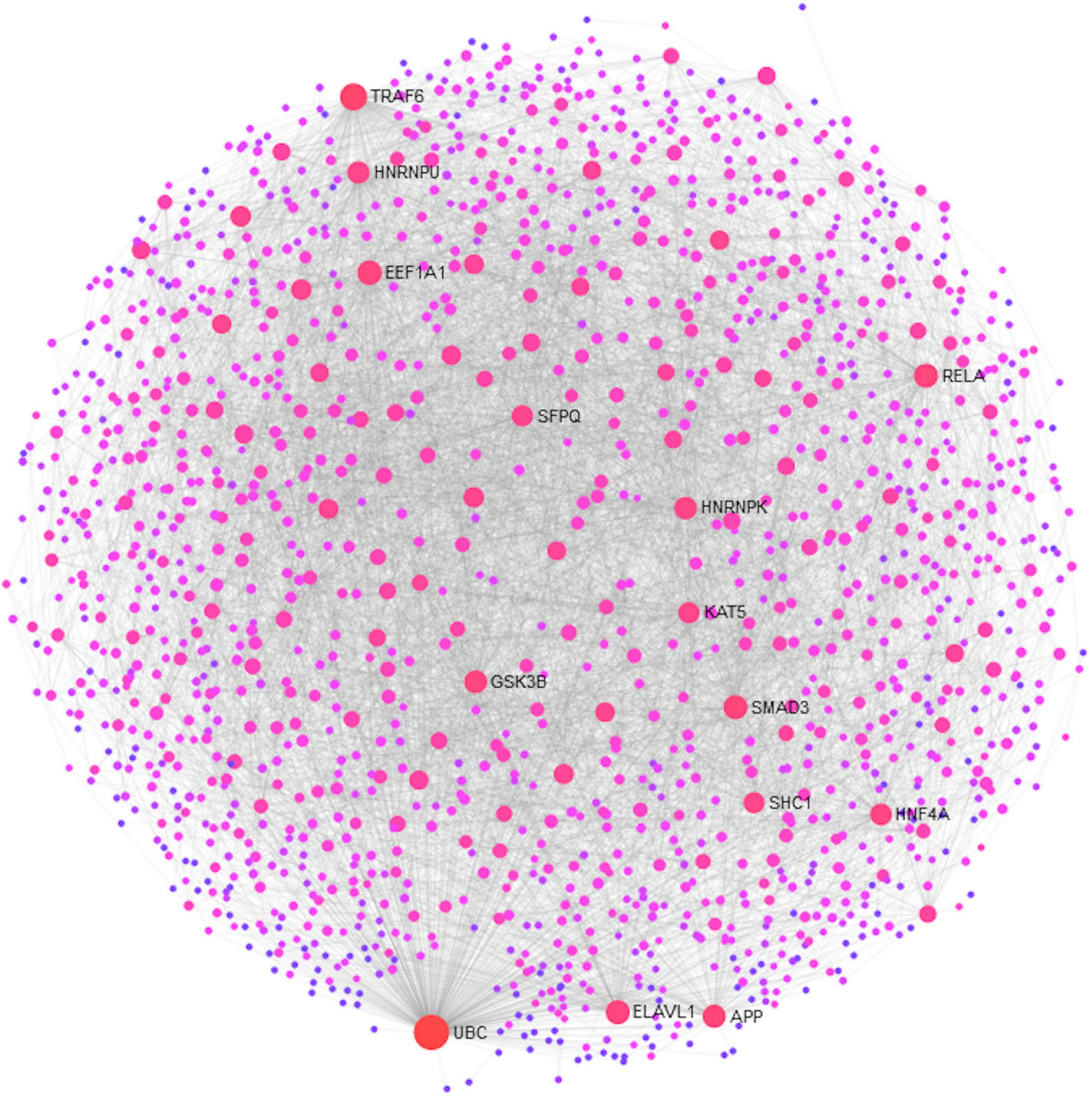
Protein-protein interaction (PPI) network of the differentially expressed genes in MM and CM patients. The size of the nodes is based on their degree centrality, whereas the intensity of their colour reflects their betweenness centrality

The products of those genes, whose expressions differed in the MM and SM groups based on the multi-way ANOVA, were further mapped onto a human protein-protein network, leading to subnetworks. Figure 5 shows the protein-protein interaction (PPI) network of significantly regulated genes in MM, CM, and NCM patients, whereas Fig. 6 shows the PPI network of significantly regulated genes in MM and CM patients. The PPI network of genes regulated in MM, CM, and NCM patients was composed of 810 nodes including 322 seed nodes and 2574 edges, whereas the PPI network of genes regulated in MM and CM patients was composed of 1479 nodes including 629 seed nodes and 6479 edges. Additional file 4: Table S3 shows the list of the nodes and their degree and their betweenness centrality measures. When comparing the two PPI networks for the nodes with the highest degrees, the following nodes belong to the best 10 nodes in both networks: UBC, GSK3B, EEF1A1, RELA, APP, and ELAVL1. Noticeably, GSK3B is involved in KEGG pathways, such as TCR- and BCR-signalling and Alzheimer’s disease pathways. RELA is involved in TCR-, BCR-, TLR-, and RIG-I-like receptor-signalling pathways, whereas APP is involved in Alzheimer’s disease pathway. The two PPI networks were significantly enriched for more than 100 Reactome and KEGG pathways (Additional file 5: Table S4). Tables 6 and 7 shows a selection of pathways of interest, such as Alzheimer’s disease, BCR-, TCR-, TLR-, cytokine-, FcεRI-, FCGR-, RIG-I-like receptor-signalling pathways, and natural killer cell mediated cytotoxicity.

**Table 6:** Reactome and KEGG pathways enrichment analysis for genes differentially expressed between MM and CM patients

Pathways Reactome database^a^	Total	Expected	Hits	*P* value	FDR
Immune System	1140	70,7	136	4,47E-16	6,27E-13
Signaling by the B Cell Receptor (BCR)	199	12,4	44	4,15E-14	1,45E-11
Constitutive Signaling by NOTCH1 PEST Domain Mutants	59	3,67	22	1,75E-12	3,51E-10
Downstream Signaling Events Of B Cell Receptor (BCR)	173	10,8	38	3,14E-12	5,50E-10
Activation of NF-kappaB in B Cells	66	4,1	22	2,37E-11	3,69E-09
Signaling by NOTCH1	74	4,6	23	4,21E-11	3,69E-09
Signaling by ERBB4	152	9,45	33	1,26E-10	9,31E-09
Cytokine Signaling in Immune system	286	17,8	44	1,24E-08	4,98E-07
ER-Phagosome pathway	63	3,92	17	1,59E-07	4,04E-06
TCR signaling	65	4,04	17	2,60E-07	5,89E-06
RIG-I/MDA5 mediated induction of IFN-alpha/beta pathways	67	4,16	17	4,19E-07	8,51E-06
Cross-presentation of soluble exogenous antigens	48	2,98	14	7,08E-07	1,34E-05
TRAF6 Mediated Induction of proinflammatory cytokines	62	3,85	16	7,18E-07	1,34E-05
Fcgamma receptor (FCGR) dependent phagocytosis	86	5,35	19	8,95E-07	1,65E-05
Platelet activation signaling and aggregation	220	13,7	33	1,64E-06	2.84E-05
DAP12 signaling	164	10,2	27	2,47E-06	4,23E-05
TRAF6 mediated induction of NFkB and MAP kinases upon TLR7/8 or 9 activation	76	4,72	17	2,82E-06	4,76E-05
Toll Like Receptor 7/8 (TLR7/8) Cascade	77	4,79	17	3,42E-06	5,51E-05
TRIF-mediated TLR3/TLR4 signaling	87	5,41	18	4,73E-06	7,21E-05
Toll Like Receptor 9 (TLR9) Cascade	79	4,91	17	4,96E-06	7,30E-05
MyD88-independent cascade	88	5,47	18	5,61E-06	8,03E-05
Toll Like Receptor 10 (TLR10) Cascade	74	4,6	16	8,84E-06	1,15E-04
Toll Like Receptor 5 (TLR5) Cascade	74	4,6	16	8,84E-06	1,15E-04
Pathways from KEGG database^a^	Total	Expected	Hits	*P* value	FDR
Neurotrophin signaling pathway	123	8,75	31	1,98E-10	7,16E-09
Bacterial invasion of epithelial cells	56	3,98	20	4,68E-10	1,45E-08
Notch signaling pathway	47	3,34	18	9,49E-10	2,06E-08
T cell receptor signaling pathway	98	6,97	25	9,42E-09	1,57E-07
Endocytosis	101	7,19	23	3,59E-07	3,71E-06
B cell receptor signaling pathway	75	5,34	19	6,75E-07	6,1E-06
RIG-I-like receptor signaling pathway	49	3,49	12	0,000115	0,000653
Leukocyte transendothelial migration	108	7,68	19	1,75E-04	8,61E-04
Epithelial cell signaling in Helicobacter pylori infection	37	2,63	10	1,79E-04	8,61E-04
Toll-like receptor signaling pathway	97	6,9	16	1,18E-03	4,64E-03
Fc epsilon RI signaling pathway	75	5,34	12	6,10E-03	2,1E-02
Alzheimer’s disease	49	3,49	8	2,08E-02	6,28E-02

^a^Pathways have selected among those that were significant

**Table 7:** Reactome and KEGG pathways enrichment analysis for genes differentially expressed between MM, CM and NCM patients

Pathways from Reactome database^a^	Total	Expected	Hits	*P* value	FDR
Activation of NF-kappaB in B Cells	66	7.06	31	7,21E-14	9,19E-12
Signaling by the B Cell Receptor (BCR)	199	21.3	58	2,40E-13	2,59E-11
Adaptive Immune System	654	69.9	128	7,82E-13	6,57E-11
Downstream Signaling Events Of B Cell Receptor	173	18.5	51	4,45E-12	2,83E-10
Signaling by Interleukins	116	12.4	39	1,92E-11	1,03E-09
Signalling by NGF	290	31	67	3,70E-10	1,18E-08
NOTCH1 Intracellular Domain Regulates Transcription	50	5.35	21	1,02E-08	2,16E-07
ER-Phagosome pathway	63	6.74	23	4,85E-08	8,09E-07
Platelet activation signaling and aggregation	220	23.5	50	1,23E-07	1,92E-06
TRAF6 mediated induction of NFkB and MAP kinases upon TLR7/8 or 9 activation	76	8.13	25	1,37E-07	2,08E-06
Toll Like Receptor 7/8 (TLR7/8) Cascade	77	8.23	25	1,82E-07	2,69E-06
MyD88-independent cascade	88	9.41	27	2,19E-07	3,17E-06
Toll Like Receptor 3 (TLR3) Cascade	88	9.41	27	2,19E-07	3,17E-06
Activated TLR4 signalling	100	10.7	29	3,02E-07	4,22E-06
Fcgamma receptor (FCGR) dependent phagocytosis	86	9.2	26	5,07E-07	6,71E-06
Toll Like Receptor 4 (TLR4) Cascade	103	11	29	6,01E-07	7,80E-06
Pathways from KEGG database^a^	Total	Expected	Hits	*P* value	FDR
T cell receptor signaling pathway	98	11,1	35	1,15E-10	5E-09
Bacterial invasion of epithelial cells	56	6,32	24	1,47E-09	4,55E-08
B cell receptor signaling pathway	75	8,46	25	2,66E-07	2,8E-06
Fc gamma R-mediated phagocytosis	97	10,9	28	1,46E-06	1,22E-05
Endocytosis	101	11,4	27	1,11E-05	6,88E-05
TGF-beta signaling pathway	84	9,48	20	8,03E-04	3,71E-03
Fc epsilon RI signaling pathway	75	8,46	18	1,31E-03	5,48E-03
Toll-like receptor signaling pathway	97	10,9	21	2,19E-03	8,64E-03
RIG-I-like receptor signaling pathway	49	5,53	12	6,85E-03	2,25E-02
Alzheimer’s disease	49	5,53	11	1,82E-02	5,41E-02
Natural killer cell mediated cytotoxicity	138	15,6	23	3,41E-02	9,37E-02

^a^Pathways have selected among those that were significant

## Discussion

In this study, we investigated blood gene expression patterns in patients with MM, severe NCM, or CM. We identified a molecular signature associated with CM in humans, which was enriched with pathways involved in the activation of immune cells, such as monocytes, macrophages, B and T lymphocytes, and natural killer cells.

We performed microarray experiments and analysed the data using one-way statistical analyses. We identified genes whose expression levels differed at a nominal *P* value level under 0.001. However, no significant genes remained after multiple test corrections. Since several confounding factors may diminish the power of the analysis, we assessed the effects of age, gender, and leucocyte count on gene expression, and further took their effects into account. Also, we identified 443 genes, the expressions of which differed in MM, severe NCM, and CM patients. This molecular signature mainly discriminated the CM patients from the others; in the same way, severe anaemia and CM patients were reported to differ in their transcriptional and immunological profiles [24, 25]. Furthermore, we focused on CM patients in comparison to MM patients, and identified 842 genes, the expressions of which differed in MM and CM patients. These results are in line with those obtained in blood and brain samples from CM mice [7, 26], and support the existence of blood gene expression profiles associated with CM. Furthermore, they suggest that gene expression profiles might be detected before the occurrence of CM in humans. Such predictive profiles have been identified in mice with CM [7].

Functional enrichment analyses identified several pathways that may be altered by the regulation of gene expression in CM patients. Interestingly, we detected an enrichment of a neurodegenerative disease signature, as previously reported [13, 24], and we identified APP, which is involved in Alzheimer’s disease [27], as a node with a high number of interactions within the PPI network associated with CM. This suggests that some biological pathways are involved in both CM and neurodegenerative diseases. Nevertheless, most of the identified pathways were immune pathways. Strikingly, the functional enrichment of molecular signatures in specific cells using MSigDB data was consistent with the functional enrichment of PPI networks, which were constructed on the basis of regulated genes. These included BCR-, TCR-, TLR-, FcεRI-, FcγR-, and RIG-I-like receptor- signalling pathways, and natural killer cell mediated cytotoxicity pathway. Noticeably, BCR- and TCR- signalling pathways were also associated with CM, on the basis of the brain transcriptional analysis of CM-resistant and CM-susceptible mice [7], whereas the TLR-signalling pathway was associated with SM in Mali [12]. Moreover, there was an over-representation of MSigDB molecular signatures of activated monocytes, dendritic cells, macrophages, NK cells, B lymphocytes, CD8 T lymphocytes, and CD4 T lymphocytes, including Th1, Th2, and T regulatory cells. These findings are consistent with the involvement of those immune cells in malaria pathogenesis. IgG produced by B lymphocytes activate effector cells through FcγRIIA to kill the parasites [28]. Monocytes and macrophages activated through TLR and Th1 effector lymphocytes produce pro-inflammatory cytokines that are thought to be involved in both parasite clearance and immunopathology [29]. Pro-inflammatory cytokines, such as TNF and IFNγ, increase the expression of adhesion molecules on endothelial cells, such as ICAM1, leading to the binding of infected red blood cells on endothelial cells [30–32]. It has been suggested that histamine-secreting basophils activated by IgE through FcεRI increase vaso-permeability and the over-expression of ICAM1 on endothelial cells, suggesting that Th2 lymphocytes promoting the production of IgE play a role in malaria [33]. Besides, IFNγ that is produced by Th1 lymphocytes induces the expression of CXCL10 by endothelial cells, leading to an increased adhesion of T lymphocytes [31]. CD8 T lymphocytes recognize plasmodial antigens presented by the MHC class I of the endothelial cells, leading to the death of endothelial cells, to the disruption of the blood-brain barrier, and, potentially, cerebral haemorrhages, as shown in mice with CM [34–36]. T regulatory lymphocytes are thought to be the key actors of Th1 effector lymphocyte regulation in malarial infection, and an excessive T regulatory induction, which might lead to high parasitaemia at the early stage of infection, might benefit the host at later stages by preventing an excessive Th1 effector lymphocyte response [37], including an excessive production of pro-inflammatory cytokines. In this way, *IL18R* and *IL1RN* that encode for receptors of pro-inflammatory cytokines were up-regulated in malaria patients in Benin [38] and in CM patients in our study population, whereas *IL18R* was up-regulated in blood from CM mice [26]. Interestingly, IL18 produced by myeloid cells is required for an optimal activation of NK cells upon contact with infected red blood cells [39]. The role of NK cells remains, nevertheless, to be clarified. Although NK cells control malaria infection by killing the parasite and by early producing IFNγ, NK cells may produce inflammatory cytokines in brain microvessels and may amplify the sequestration of infected red blood cells and CD8 T lymphocytes and the disruption of the blood-brain barrier [34, 40]. However, patients with SM were reported to have a lower NK cell cytotoxicity activity than patients with MM, suggesting a protective role against SM [41]. In addition, the RIG-I-like receptor-signalling pathway, which was enriched in genes regulated in CM patients in our study, was shown to be an important pathway to activate the NK cells and kill the parasite [41].

Although our results are in line with published results and propose signal transduction pathways involved in the pathology, they will not provide definitive insights into molecular and cellular mechanisms of cerebral malaria pathogenesis. Also, it is important to point out the limits of the study. First, transcriptional profiling may be influenced by the differences in cellular composition in PBMC. Furthermore, looking for gene expression profiles in well defined cell types will likely provide specific transcriptional profiles associated with CM. Using single-cell RNA sequencing approach would be a promising approach to reach this objective [42]. Second, the sample size of our study is small, and replication studies should be performed. Third, studying blood cells gives an incomplete view of CM pathogenesis mechanisms, because other cells and tissues are involved. In particular, brain microvascular endothelial cells play a central role in CM [30, 31, 43, 44]. However, obtaining brain endothelium in humans is very difficult, and in vitro and in vivo models are rather used to investigate the role of such cells in CM. Fourth, it is not possible to determine the molecular factors causing CM on the basis of our results, because gene expression levels were measured at the time of malaria attacks. Measuring gene expression levels in patients before CM attacks is very challenging. Interestingly, such studies that have been performed in mice infected by *Plasmodium berghei* ANKA led to identify predictive profiles before the onset of cerebral malaria [7]. This suggests that new candidate causal factors can be identified in this in vivo model and can be tested using gene targeting approach in mice infected by *Plasmodium berghei* ANKA. It should be stressed, however, that some authors wrote that the murine model is not relevant for the human disease, whereas other authors pointed out that there are many common features between mouse pathology and human pathology [30, 31, 44, 45]. In particular, White et al. maintained that mice with CM show no or little parasite sequestration within the brain, and that human CM mainly results from parasite sequestration and brain microvessels obstruction [46]. Interestingly, Strangward et al. recently showed that the accumulation of parasitized red blood cells in brain microvessels was a feature of mouse CM that was not observed during uncomplicated malaria attacks [47]. It is likely, however, that a single mouse model does not fully reflect the biological mechanisms underlying CM in humans. Noticeably, there is accumulating evidence of the critical role of CD8+ T lymphocytes in mouse CM, whereas there is little evidence of high numbers of CD8+ T lymphocytes in human brain microvessels during CM attacks. Brain endothelial cells have been shown to crosspresent *Plasmodium berghei* ANKA antigens to mouse CD8+ T lymphocytes [34, 35], whereas it has not been reported in humans. Nevertheless, a human brain endothelial cell line has been shown to phagocytose *P. falciparum* merozoites, suggesting that human endothelial cells may crosspresent plasmodial antigens to human CD8+ T lymphocytes [35]. Also, we consider that the mouse CM model can provide new functional hypotheses that should be tested in humans.

## Conclusions

We identified genes expression profiles that discriminated between CM and MM patients on the one hand, and immune molecular pathways associated with CM on the other hand. Our results are in line with the involvement of monocytes, macrophages, dendritic cells, and B and T lymphocytes, and NK cells in malaria pathogenesis. Further studies are required to confirm the molecular signatures of CM, and to identify those of severe NCM. In addition, further comparisons of severe malaria patient gene expression levels to that of mild malaria patients may reveal a molecular signature common to severe malaria phenotypes. Finally, genetic variation that influences gene expression in patients with clinical malaria may modulate the immunopathological response. We think that looking for such regulatory variants will help to better uncover the genetic basis of severe forms of malaria in African populations.

## Supplementary information

**Additional file 1: Table S1.** Differentially expressed genes in MM vs CM vs NCM and MM vs CM analyses. Results of the Welch t test, one-way ANOVA analyses, and multi-way ANOVA analyses are shown.

**Additional file 2: Figure S1.** The correlation between gene expression levels measured by qPCR and those measured by microarray technology for CM and MM patients. Eight genes were selected. The qPCR data were analysed by the 2^-deltadeltaC(t)^ method with GAPDH as a control gene. Both the qPCR and the microarray data were normalized on the basis of the values obtained with a reference sample.

**Additional file 3: Table S2.** Enriched MSigDB signatures for MM vs CM vs NCM and MM vs CM analyses.

**Additional file 4: Table S3.** Protein-protein interaction (PPI) network nodes: degree and betweenness centrality for MM vs CM vs NCM and MM vs CM analyses.

**Additional file 5: Table S4.** Enriched Reactome and KEGG pathways for MM vs CM vs NCM and MM vs CM analyses.

## Data Availability

Data are either included in the published paper or available in the GEO database under GSE116306.

## Abbreviations

CM: Cerebral Malaria
FDR: False Discovery Rate
MM: Mild Malaria
NCM: Non-Cerebral Malaria
PBMC: Peripheral Blood Mononuclear Cells
PPI: Protein-Protein Interaction
RNA: RiboNucleic Acid
SM: Severe Malaria
SVM: Support Vector Machine
