# Supplementary figures and images for: Gene expression profiling in blood from cerebral malaria patients and mild malaria patients living in Senegal
Source: BMC Med Genomics. 2019 Oct 30;12:148. doi: 10.1186/s12920-019-0599-z (PMC6821028; doi:10.1186/s12920-019-0599-z)

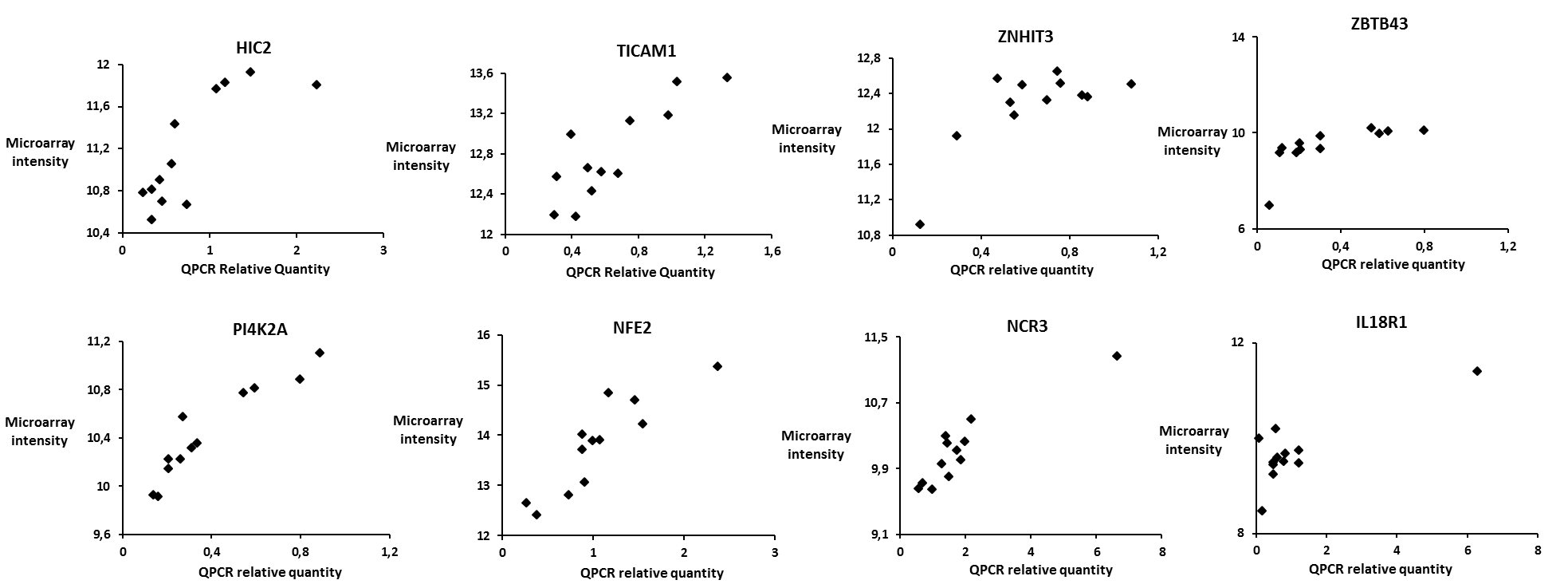

Supplement: Supplementary file 2 — Additional file 2: Figure S1. The correlation between gene expression levels measured by qPCR and those measured by microarray technology for CM and MM patients. Eight genes were selected. The qPCR data were analysed by the 2-deltadeltaC(t) method with GAPDH as a control gene. Both the qPCR and the microarray data were normalized on the basis of the values obtained with a reference sample. [file 12920_2019_599_MOESM2_ESM.tif]
